# Relationships of climate, human activity, and fire history to spatiotemporal variation in annual fire probability across California

**DOI:** 10.1371/journal.pone.0254723

**Published:** 2021-11-03

**Authors:** Isaac W. Park, Michael L. Mann, Lorraine E. Flint, Alan L. Flint, Max Moritz

**Affiliations:** 1 Department of Ecology, Evolution, and Marine Biology, University of California–Santa Barbara, Santa Barbara, California, United States of America; 2 Department of Geography, George Washington University, Washington, D.C., United States of America; 3 U.S. Geological Survey, Sacramento, CA, United States of America; 4 University of California Cooperative Extension, Santa Barbara, CA, United States of America; 5 Bren School of the Environment, University of California–Santa Barbara, Santa Barbara, CA, United States of America; University of British Columbia, CANADA

## Abstract

In the face of recent wildfires across the Western United States, it is essential that we understand both the dynamics that drive the spatial distribution of wildfire, and the major obstacles to modeling the probability of wildfire over space and time. However, it is well documented that the precise relationships of local vegetation, climate, and ignitions, and how they influence fire dynamics, may vary over space and among local climate, vegetation, and land use regimes. This raises questions not only as to the nature of the potentially nonlinear relationships between local conditions and the fire, but also the possibility that the scale at which such models are developed may be critical to their predictive power and to the apparent relationship of local conditions to wildfire. In this study we demonstrate that both local climate–through limitations posed by fuel dryness (CWD) and availability (AET)–and human activity–through housing density, roads, electrical infrastructure, and agriculture, play important roles in determining the annual probabilities of fire throughout California. We also document the importance of previous burn events as potential barriers to fire in some environments, until enough time has passed for vegetation to regenerate sufficiently to sustain subsequent wildfires. We also demonstrate that long-term and short-term climate variations exhibit different effects on annual fire probability, with short-term climate variations primarily impacting fire probability during periods of extreme climate anomaly. Further, we show that, when using nonlinear modeling techniques, broad-scale fire probability models can outperform localized models at predicting annual fire probability. Finally, this study represents a powerful tool for mapping local fire probability across the state of California under a variety of historical climate regimes, which is essential to avoided emissions modeling, carbon accounting, and hazard severity mapping for the application of fire-resistant building codes across the state of California.

## Introduction

Variation in fire activity arises from patterns of local vegetation, climate, and ignitions on the landscape. Wildfire thus requires convergence of these factors in the ‘fire regime triangle’, respectively involving gradients of sufficient flammable resources, conditions that are conducive to propagation of fire, and influences on how fires start and stop. All of these factors are variable across space and time, and can be mediated by multiple aspects of local conditions including precipitation, fire weather patterns, human activity, and the length of time since the most recent fire event [1–4]. Previous examinations by Westerling & Bryant [5], for example, found nonlinear relationships between many aspects of local climate and fire probabilities throughout California. Similarly, the relationship between burned area and local population density, a critical metric for human activity, has been documented to exhibit a non-monotonic relationship in California [6], and varies globally across climate regions, vegetation regimes, and local land use [7].

Human activity is well documented to play a dominant role not only in fire ignition, but also suppression, land fragmentation, and in some cases, the quantity of flammable resources throughout California [6, 8, 9], particularly in Mediterranean-climate ecoregions [9]. Although changes in human-induced ignitions may not translate directly into increases in area burned or in the resultant fire probability at any location [10], human activity is important in predicting fire probability and burned area throughout California at broader scales [11]. For instance, burned area typically increases in tandem with population density and anthropogenic ignitions in moderately populated areas, but decreases in highly populated areas due to a reduction in fuel continuity and greater management effort towards preventing wildfires [2, 12, 13]. California encompasses both densely populated urban centers and large tracts of largely uninhabited wildlands, as well as many degrees of habitation in between. Thus, it is critical that we determine the precise nature of these relationships and their potential for nonlinearity to accurately assess current and future fire probabilities across California’s diverse fire regimes [11].

Variations in local climate also play a major role in determining local fire probability by altering the quantity and structure of local fuels [14, 15], as well as the dryness of fuel and the length of the fire season [16–18]. Increasing drought and warming temperatures have been associated with larger fire size [19, 20], stronger burn intensity, and more rapid rates of spread by wildfire. This has led to greater suppression difficulty and shortened fire intervals throughout much of California [21–24]. Examination of the Rim Fire, a large wildland fire that occurred in the California Sierra Nevada in 2013, found that local water balance (as measured by climatic water deficit [CWD] and actual evapotranspiration [AET]) is an effective predictor of both area burned and burn severity [25]. Moreover, CWD was found to be the most effective predictor of fire distribution surrounding Lake Tahoe, regardless of whether the ignitions were due to lightning or human activity [26]. However, CWD and AET reflect quite different aspects of local conditions as relates to vegetation and wildfire occurrence. CWD, which measures the degree to which evaporative demand exceeds available soil moisture, can be viewed as a proxy for conditions favorable for vegetation flammability [1]. AET, in contrast, may be seen as a proxy for vegetation productivity, vegetative biomass, and regrowth [1, 27]. In addition to long-term climate conditions, shorter-term climate variations have been found to play a significant role in rates of wildfire occurrence throughout the Western United States [28]. Positive annual AET anomalies have been associated with increased area burned in some ecosystems [27]. Similarly, wildfire frequencies throughout both northern and southern California have been positively associated with wetter than average conditions over the preceding three years due to the ability of wet conditions to spur additional plant growth and fuel buildup [29, 30]. However, the effects of these drivers on wildfire probabilities can be complex, and have been observed to interact with ignitions and other anthropogenic factors [31]. To better capture the dynamics of wildfire on the landscape, it is therefore clear that both human activity and climatic factors must be taken into account when aiming to predict the probability of fire across the landscape.

In addition to the effects of local climate and human activity on fire, the amount of time since the most recent fire may mediate the probability of fire at any given location. It is widely acknowledged that after each fire event, a period of time must elapse for local vegetation to regenerate sufficiently to sustain another wildfire. In many ecosystems, flammability (and therefore the probability of wildfire) may be strongly reduced in the years immediately following a fire event due to post-fire fuel limitations, which can reduce the size, severity, and probability of subsequent burns until vegetation has sufficiently regenerated [32–34]. However, the actual importance of stand-age in determining local fire probability throughout many California ecosystems remains uncertain and may vary both across vegetation types and among ecoregions [32, 35–37]. Further, post-fire succession produces complex shifts in the composition of the local vegetation that may alter the quantity, structure, and flammability of both live vegetation [38] and dead fuels [39] in complex ways. As a result, the relationship between annual fire probability and the time since last fire may be nonlinear, complex, and contingent on local climate [11, 12].

Concerns were raised that the broad-scale modeling of fire probabilities at statewide scales may not be possible without the use of regional sub-models due to the extremely heterogeneous nature of climate and vegetation regimes in California. Previous studies have determined that both the magnitude and nature of the relationship between local conditions and fire probabilities may differ widely among distinct ecoregions [36, 40], along aridity gradients [14], and among vegetation types [41] throughout California. Thus, regional models may capture different relationships for each independent variable. However, multiscalar examinations of fire risk from local through continental scales found that, while the predictive power of localized models sometimes remained high when extrapolated to novel regions, such models typically performed poorly because local conditions often exhibited little overlap with regions in which models were trained [42]. Thus, these apparent regional differences in the spatiotemporal relationship between local conditions and fire may reflect shifts that occur in consistent ways as local conditions change over space. If so, observed relationships between local climate and fire probability within each region may each simply represent a portion of the overall nonlinear relationship between local climate and fire probability across a wider climate gradient. Broad-scale models, which encompass a wider array of conditions, may therefore be more capable of capturing these underlying relationships than localized models in which the degree of variation in local climate or human activity is typically limited. Localized modeling also has several limitations and potential pitfalls in comparison to models constructed across broader areas. As localized models typically incorporate both a smaller number of fires and a more limited range of climate conditions, they may be both more prone to overfitting due to the limited number of specific fire events available in the training dataset, and also less capable of predicting fire probability under conditions that are rarely encountered within that region (e.g., most commonly encountered at the edges of ecoregions where conditions transition into alternate vegetation and climate regimes). By incorporating nonparametric estimators into broad-scale models of fire probability, it may be possible to avoid these limitations while both (a) incorporating contextual shifts in the importance of each parameter to fire across the conditions within each local region, and (b) revealing the underlying relationships to fire that persist across a range of local conditions and spatial scales.

For this study, we applied a GAM (Generalized Additive Model) framework to examine annual fire probabilities across California from 1970–2016. GAMs have previously been used to successfully model the presence and absence of fire across California at longer time scales [43]. This study refines these methods to predict fire probability at annual intervals by incorporating both short- and long-term climate variations, additional temporally dynamic parameters assessing different aspects of human activity on the landscape, and the time elapsed since the previous fire event within each pixel and year. We use GAMs to address two open questions in fire science. First, we tested the hypothesis that human activity will have equal or greater influence on local annual fire probabilities than local climate conditions. Second, we tested the hypothesis that statewide models of annual fire probability will outperform localized regional models (based in this study on local California ecoregions). We addressed these hypotheses with a series of statewide and regional models of annual fire probability developed using past conditions throughout California from 1970 through 2016 at 1-km scales. Additionally, we determined the potential for nonlinear modeling frameworks to provide robust predictions of annual fire probability while simultaneously revealing the underlying factors driving observed patterns of fire throughout California.

## Materials and methods

In this study, we developed a method for estimating annual fire probability from 1970–2016 throughout the state of California at a 1000-meter spatial resolution using a GAM framework. To capture both long-term conditions and interannual variability, we incorporated both 1951–1980 climate normals and the mean deviation from these normal conditions over the three years preceding each year of interest (i.e. each year under examination). Three-year deviations from climate normals were used in preference to annual deviations as a compromise between incorporating conditions in the year of interest and the documented effects of climate conditions in the years preceding the year of interest on wildfire [44]. This coincides with an ecologically meaningful period, as wet conditions over the preceding three years have previously been associated with greater wildfire frequencies throughout California [29, 30]. In addition, this model incorporated multiple aspects of human activity and development on the landscape, including local housing density, distance from roads or electrical infrastructure, and the proportion of the local area under agricultural cultivation.

Our GAM framework, while appropriate for predicting probability across broad areas, does not attempt to mechanistically model the ignition, progression, or intensity of any specific fire event. Instead, the GAM framework provides spatially explicit predictions of fire probability across the California landscape in order to contextualize management practices and to project future fire probabilities under variable climate and land use regimes. Further, to compare how statewide versus regional models influence the accuracy of predicted fire probability, we used data at the state level and for each CalVeg ecosystem province [45], which we later explain in more detail.

### Data sources

Climate data used in this study was drawn from the California Basin Characterization Model v8 [46, 47], and consists of monthly estimates of cumulative water deficit (CWD) and actual evapotranspiration (AET) from 1951–2016. This dataset represents a 270-m grid-based model of water balance calculations that incorporates climate inputs through PRISM data [48] in addition to solar radiation, topographic shading, cloudiness, and soil properties to estimate evapotranspiration [49]. Using these monthly values, we calculated the 1951–1980 mean CWD and AET normals, as well as mean deviations from those normals over a three-year period preceding each year of interest.

**Fig 1:**
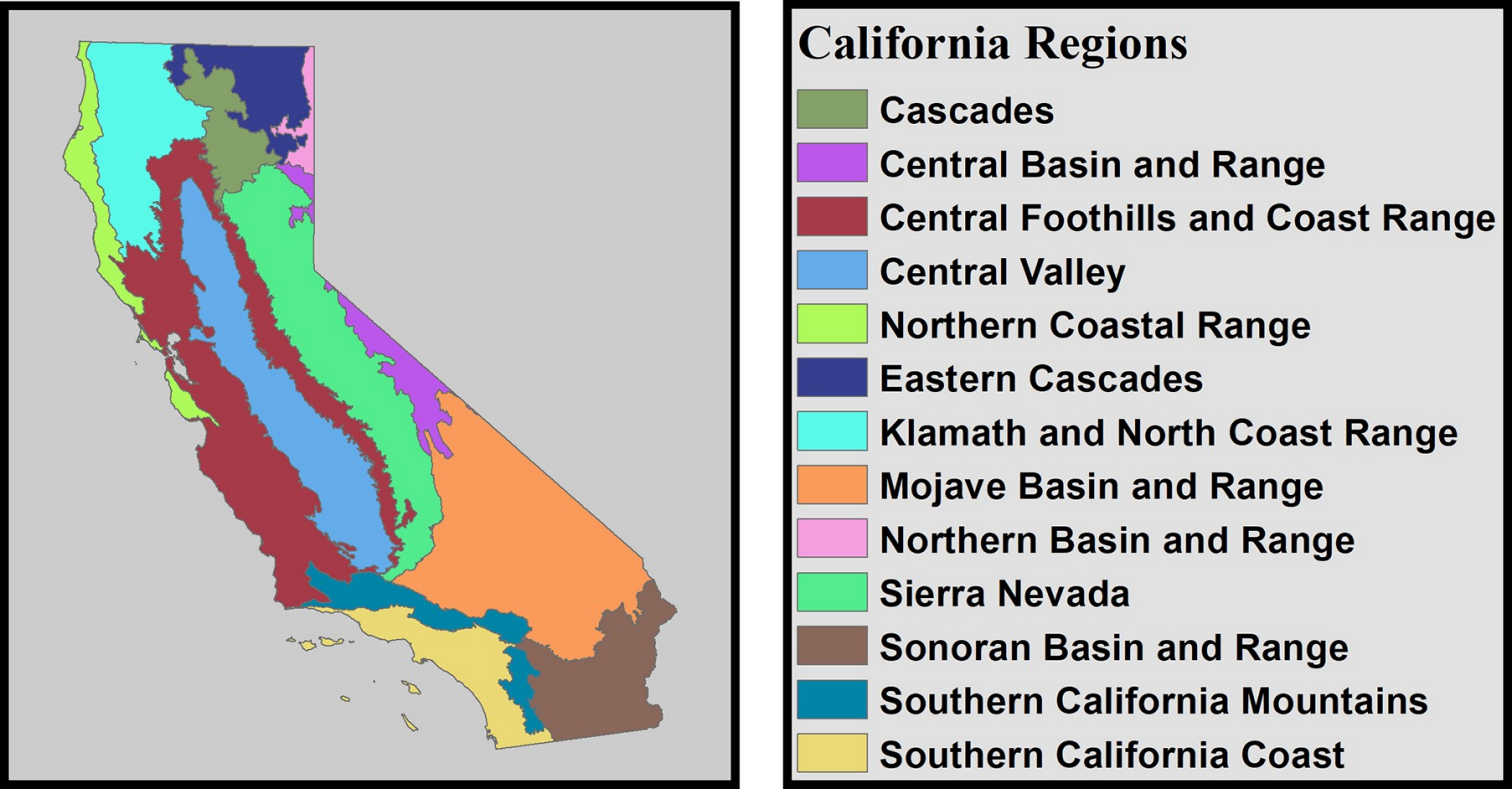
Regions of California, corresponding to CalVeg ecosystem provinces [45].

Cultivated and agricultural areas were identified using the 2016 National Land Cover Database data [50], which estimated dominant land cover throughout North America at 30-m resolution. The proportion of cultivated area and of water features that covered each 1-km pixel were then calculated by resampling to 1-km scale. Mean housing density data was drawn from the Integrated Climate and Land-Use Scenarios (ICLUS) dataset [51], which provides decadal estimates of housing density throughout the United States from 1970–2020. As precise continuous estimates of housing density were not available, housing density within each pixel was set to the mean of its class. Annual values were estimated from decadal data using linear interpolation. Ecoregions within California (hereafter referred to as “regions”) were delineated using CalVeg ecosystem provinces data [45] (Fig 1).

Road data were drawn from 2018 TIGER layer data, and consisted of all primary and secondary roads across California [52]. Electrical infrastructure data was drawn from 2020 transmission lines data [53]. In both cases, the distance of nearest roads or transmission lines to each pixel were then calculated. Pixels which contained roads or electrical infrastructure were assigned distances of 0 km.

Fire history data was drawn from FRAP fire perimeter data [54], which incorporates perimeters of all known timber fires >10 acres (>0.04 km^2^), brush fires >30 acres (>0.12 km^2^), and grass fires >300 acres (>1.21 km^2^) from 1878–2017. Using this data, the presence of fire in each 1-km pixel was classified in a binary fashion (e.g. 1 for burned, 0 for unburned) for each year of interest. Due to computational limits and the quantity of data involved in this study, we did not calculate burned area within each pixel, or distinguish pixels in which a single fire occurred in a given year from those in which multiple fires occurred. This data was also used to calculate the number of years since the most recent fire within any pixel, prior to each year in which fire probability was projected. Thus, locations in which no fire was observed throughout the fire record were treated as having gone a maximum of 100 years without a fire event for the purposes of model construction. These pixels comprised 29% - 33% of data annually (depending on year), and included both locations in which fire would not be expected (such as highly xeric regions) as well as locations in fire-prone areas in which no fire had been documented within the FRAP fire perimeter data used in this study.

### Data preparation

To manage computational load, we resampled all datasets to 1-km pixels using Rasterio in Python v3.7 [55]. In the case of cultivated lands and water features, the percent area covered by those features within each resulting 1-km pixel was calculated from the 30-meter national land cover data. Each dataset was masked to exclude locations outside California state boundaries, pixels in which 50% or more of the area was characterized by water features according to the 2016 National Land Cover Dataset [56], and pixels in which BCM climate data was not available (S1 Fig). To produce a training dataset of manageable size, we further subsampled these data using Poisson-disk sampling [57]. This method allows the selection of randomly distributed pixels across a surface while ensuring a minimum distance threshold between selected pixels, thereby minimizing the likelihood of clumped samples and the resulting issues arising from spatial autocorrelation among pixels that were selected in close proximity (S1 Fig, Table 1). In this study, a minimum distance of 5 km was maintained among all selected pixels used to train statewide and regional models, resulting in a total of 493,876 pixels selected across California. This threshold represents a tradeoff between ensuring a high number of pixels and observed fire events available for model training, while also restricting the dataset to a computationally manageable size (S1 Table).

**Table 1:** Description of variables used to estimate annual fire probability.

Variable		Description	Time Variant
**Climate**			
	Actual Evapotranspiration (AET) Normal	1951–1980 mean annual actual evapotranspiration (mm)	True
	Actual Evapotranspiration (AET) Deviation	Mean 3-year Deviation from 1951–1980 mean annual actual evapotranspiration normal (mm)	True
	Climatic Water Deficit (CWD) Normal	1951–1980 mean annual climatic water deficit normal (mm)	True
	Climatic Water Deficit (CWD) Deviation	Mean 3-year Deviation from 1951–1980 mean annual climatic water deficit normal (mm)	True
**Human Activity**			
	Mean Housing Density	Mean housing density within a 25-km radius (Units/ha)	True
	Proportion Cultivated Area	Proportion of 1-km pixel characterized by cultivated lands	False
	Distance to Roads	Distance to paved roads (km)	False
	Distance to Electrical	Distance to electrical infrastructure (km)	False
**Other**			
	Years Since Fire	Years since most recent fire	True

### Fire probability calculation

Fire probability within each year was calculated using a binomial GAM conducted using penalized cubic regression splines in the R package MGCV [58] and integrated with additional Python code using rpy2 [59]. Training data used to predict fire probability within each year consisted of data from all training pixels selected using Poisson-disk sampling, and from all years excluding the year currently under prediction. To minimize computational time while allowing for nonlinear relationships between fire probability and each observed aspect of local conditions, a maximum of five smoothing terms was allowed for each parameter. The contribution of high and low values of each parameter to predicted fire probability were evaluated using smoothing curves for each parameter throughout the entirety of the observed parameter space. To visualize the typical contribution of each parameter to fire probabilities (excepting short-term deviations from climate normals) over space, we mapped the smoothed coefficient associated with each parameter using the 1951–1980 climate normal within each pixel across the state of California.

### Evaluating model performance under novel spatial and temporal conditions

To determine the ability of this GAM framework to predict fire probabilities both (a) in novel locations and (b) in years not present in the training dataset, model performance was assessed using multidimensional k-fold cross-validation. To accomplish this, all data were divided by year into one of ten randomly assigned temporal groups of equal size, and all pixels (of those previously selected by the Poisson-disk mask) were similarly divided into ten randomly assigned spatial groups of equal size. GAMs were then constructed iteratively while holding out one temporal and one spatial group as testing data within each iteration. The ability of these models to successfully separate high-fire probability conditions from low-fire probability conditions was evaluated by calculating the ROC/AUC score of pixels and years not included in the training dataset during each iteration. The ROC/AUC score is a performance measurement for classification problems that evaluates the degree to which the two classes (in this case burned and unburned locations in a given year) can be separated by a given model, with scores ≤ 0.5 indicating no separation between classes by the model, and scores of 1.0 indicating perfect separation [60]. Overall model performance was thus reflective of the ability of a given model permutation to predict fire probabilities in both years and locations that were novel to the data on which it was trained. This metric was preferred to other classification metrics such as balanced accuracy, recall, or F1 scores [61] because the annual probability of fire in any location was not expected to exceed 50% in any case. As a result, predicted binary classifications were expected to be zero (i.e. no fire) in all cases. Other metrics that accounted for predicted probabilities of a positive event, such as log-loss or Brier scores were also found to be inappropriate, due either to the unbalanced nature of the annual fire occurrences versus absences, which lead to biases in log loss scores, or to the comparative rarity of fire events, which limit the utility of Brier scores [62, 63]. Thus, ROC/AUC scores, which were resilient to these issues [64] were selected as the most appropriate metric for evaluating model performance.

We applied similar methods to evaluate whether localized models of fire probability outperformed models constructed using data distributed across all of California. To accomplish this, we tested the performance of localized models in predicting fire probability within their region using identical methods to those described above. In these regional models, however, both training and testing data were restricted to pixels located within the region of interest prior to assigning randomized spatial groups for cross-validation.

To determine whether regional models provided superior predictive ability to statewide models when making predictions in a region not used in training of the statewide model, we trained statewide models in iterative fashion similar to the methods described above. However, among statewide models used for these comparisons, holdout pixels were not chosen randomly in each iteration, and instead consisted of all pixels within a given region. Thus, in each iteration, we tested model performance only within a novel region not used in model training. We then assessed whether regional models significantly outperformed these statewide models in predicting fire probabilities within each region not used in training of the statewide model. This was accomplished by testing for significant differences in ROC/AUC scores between statewide and regional models using pairwise T-tests across all model iterations. Additionally, we evaluated the degree to which predictions of 1970–2016 mean fire probabilities were correlated to observed 1970–2016 (representing the period for which predictions were produced) and 1930–2016 (representing a longer period that allowed a more robust estimation of annual fire probabilities from observed fire data) mean annual fire probabilities calculated from the observed frequency of fire events within each 1-km pixel according to FRAP fire history data [54].

### Impacts of local climate and human activity on fire probability

The contributions of local climate and human activity to local fire probability were evaluated in three ways. First, to evaluate the contribution of each parameter across the range of observed spatiotemporal variation in local conditions across California, the smoothed coefficients of each parameter were plotted using matplotlib [65]. Second, to visualize the contributions of each parameter to resulting fire probability over space, we produced raster maps of the smoothed coefficients associated with the 1970–2016 mean value of each parameter at a 1-km resolution across California. Third, to evaluate the importance of each category of local conditions (i.e. local climate conditions, human activity, and short-term deviations from long-term climate normals), we evaluated the degree to which predictions of mean annual fire probability from 1970–2016 differed when the effects of all parameters other than the parameter(s) of interest were eliminated. This was accomplished by first calculating annual fire probabilities using the parameter coefficients produced by the full GAM, while eliminating the effects of parameters not included in the subset being evaluated (e.g. climate variables). In the case of parameters that had no meaningful null value (such as climate normals), the effect of spatial variation on fire probability was eliminated by setting the climate conditions in each pixel to the mean value observed in the training set. Conversely, in the case of those parameters for which a value of zero was meaningful (e.g. mean housing density, the proportion of cultivated area, and short-term climate deviations within each pixel), the effect of spatial variation on fire probability was eliminated by setting the value of that parameter to zero. To eliminate the effects of distance-related parameters, we set their values to the maximum observed in the training dataset (as zero values would typically coincide with locations in which their influence was strongest, and no other inherent null value existed). We then evaluated the contributions of each subset of parameters to overall predictions of annual fire probability by correlating the predictions of mean fire probability produced by those sub-models (climate parameters only, human activity only, and climate normals only) to those of the full model using Pearson’s correlation coefficients.

We assessed the importance of each parameter to fire probability by evaluating the degree to which mean annual predicted fire probabilities were correlated to mean observed annual fire probabilities from 1970–2016. We also calculated predictions of annual fire probability using the same GAMs, while eliminating the effects of parameter(s) that fell within a given subcategory (i.e. local climate conditions, human activity, or short-term deviations from normal climate conditions). We then assessed the degree to which each subcategory of local conditions contributed to the predictions of annual fire probability by evaluating their predicted mean annual fire probabilities using both (a) predictions of fire probability calculated using the full model, and (b) the observed mean annual fire probability.

## Results and discussion

**Fig 2:**
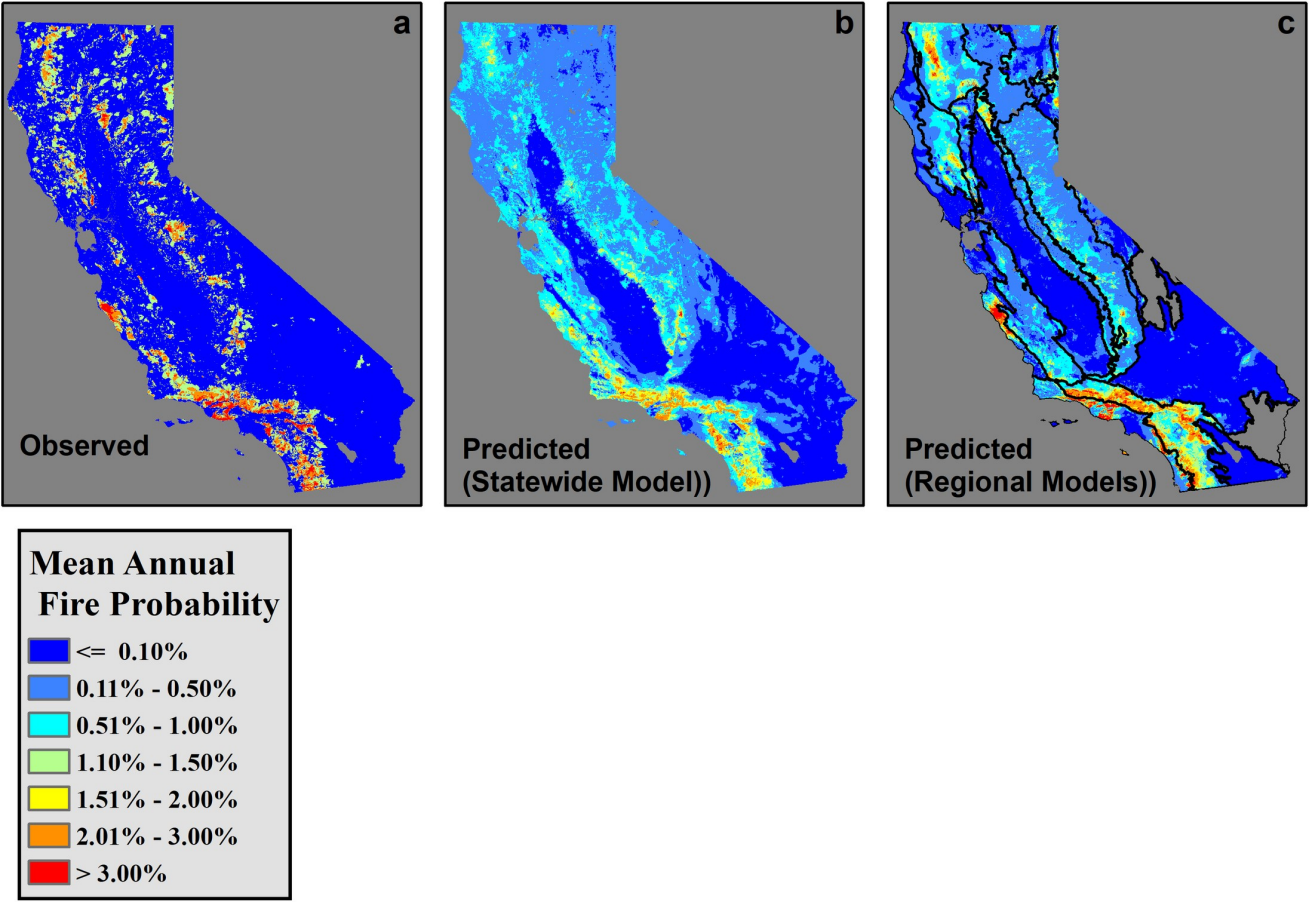
(a) Observed mean annual fire return probabilities, (b) predicted mean annual fire probabilities from 1970–2016 produced by the statewide model, and (c) by a composite of all regional models. Boundaries between regions are delineated by black lines in predictions developed by regional models.

All parameters included in this analysis exhibited significant relationships to annual fire probability in statewide models (Table 2). High ROC/AUC values also indicated that areas of high fire probability were separated successfully from those with low fire probability (ROC/AUC = 0.770, Table 3; ROC/AUC values > 0.5 and ≤ 1.0 indicate successful separation). Additionally, predictions of fire probability produced by the full model were successful in predicting the observed fire patterns (r = 0.48, p < 0.001, Table 4, Fig 2). This degree of predictive power is particularly impressive considering the limited timescale of the observed fire records and given the many locations and years in which conditions were likely primed for fire events but did not actually burn due to lack of ignition.

**Table 2:** Parameter significance of the statewide Generalized Additive Model.

Parametric Coefficient	Estimate	Std. Error	Z value	p-value
Intercept	-6.581	0.102	-64.18	<0.001
**Smoothed Parameter**	**EDF**	**Ref. df**	**Chi squared**	**p-value**
AET Normal	3.978	4	134.524	<0.001
CWD Normal	3.971	4	252.579	<0.001
AET Deviation	2.803	4	15.084	<0.001
CWD Deviation	1.873	4	5.673	0.03
Cultivated Area	3.043	4	107.926	<0.001
Mean Housing Density	3.029	4	106.490	<0.001
Years Since Fire	3.915	4	237.608	<0.001
Distance to Roads	2.549	4	11.089	0.004
Distance to Electrical	3.111	4	62.756	<0.001

The estimated degrees of freedom (EDF) for each model term indicates the potential for a curvilinear response by each term.

**Table 3:** Mean ROC/AUC across iterations of both statewide and regional models (averaged across all regions within California) in predicting fire probabilities in novel years, novel locations, and in novel years at novel locations not used in model training.

Prediction Type	ROC/AUC	
	Statewide	Regional
Novel Locations	0.767	0.716
Novel Years	0.780	0.623
Novel Locations & Years	0.767	0.624

ROC/AUC values are bounded between 0 and 1, with 1 indicating perfect model prediction.

**Table 4:** Pearson correlation between observed 1970–2016 (and 1930–2016) fire probability and predicted fire probability from 1970–2016, as well as Pearson correlation among predictions of fire probability generated using a full model, using only local climate conditions, only local climate normals, human development (consisting of local housing density, distance to electrical infrastructure, and distance to roads), cultivation (consisting of % cultivated area within each pixel), and time since the most recent fire within each pixel.

Prediction Type		Observed		Predicted
		1970–2016	1930–2016	(versus overall Model)
**All Parameters**		0.477	0.632	
**Climate Parameters Only**				
	Overall	0.302	0.353	0.696
	Climate Normal	0.305	0.356	0.998
**Human Activity Only**				
	Overall	0.396	0.565	0.762
	Development	0.405	0.605	0.742
	Cultivation	0.131	0.145	0.302
**Time Since Fire Only**				
		0.402	0.702	0.652

Correlations were significant (p <0.001, df = 403,995) in all cases.

### Effects of agriculture and human activity on fire probability

**Fig 3:**
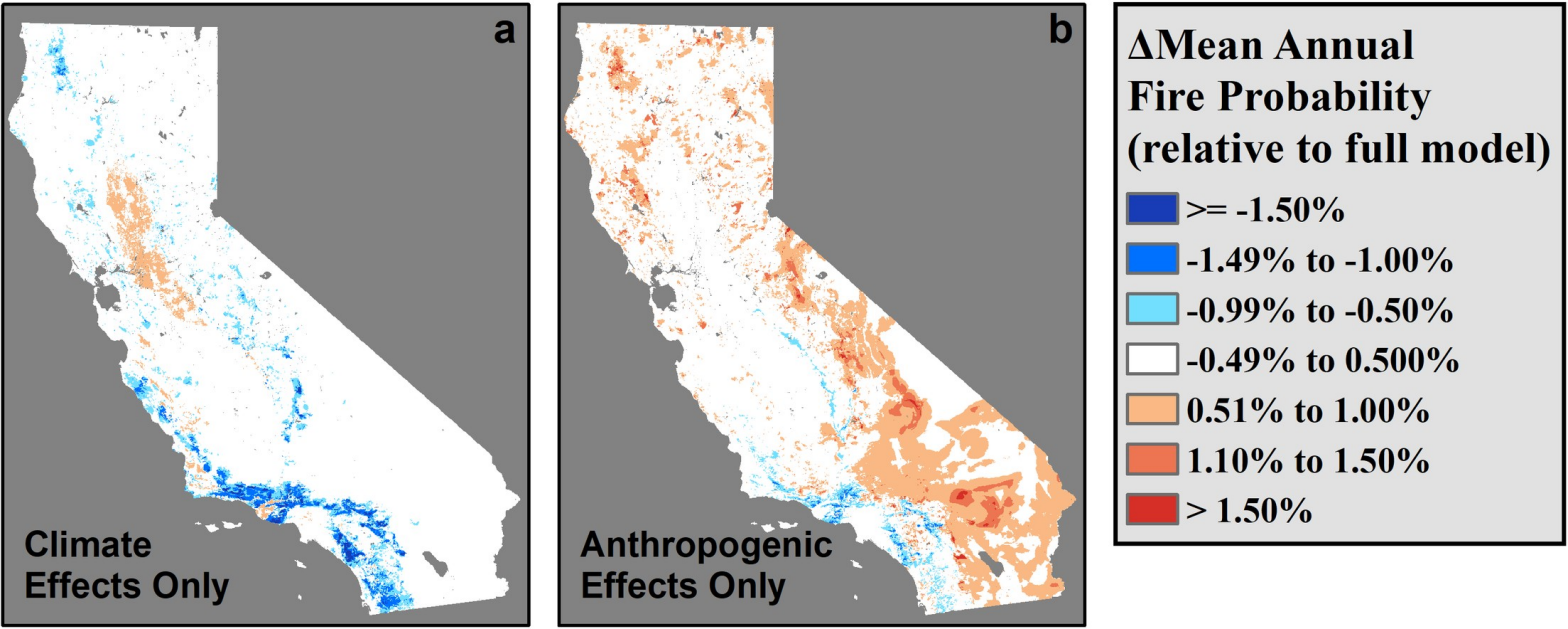
Predicted changes in mean annual fire probability after (a) eliminating the effects of human activity and after (b) eliminating the effects of variation in climate conditions throughout California.

**Fig 4:**
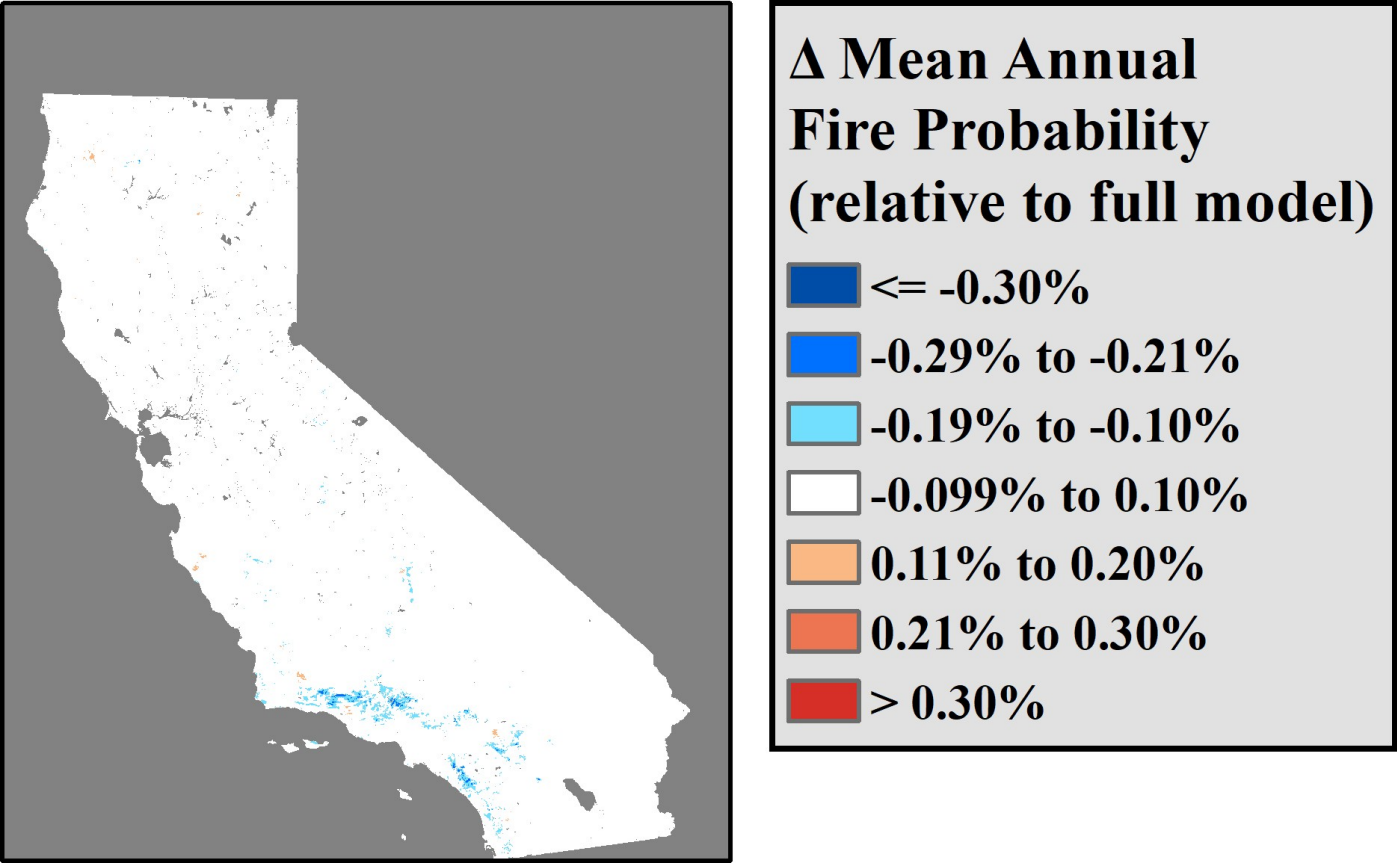
Predicted changes in 1970–2016 mean annual fire probability after eliminating the effects of short-term climate deviations.

**Fig 5:**
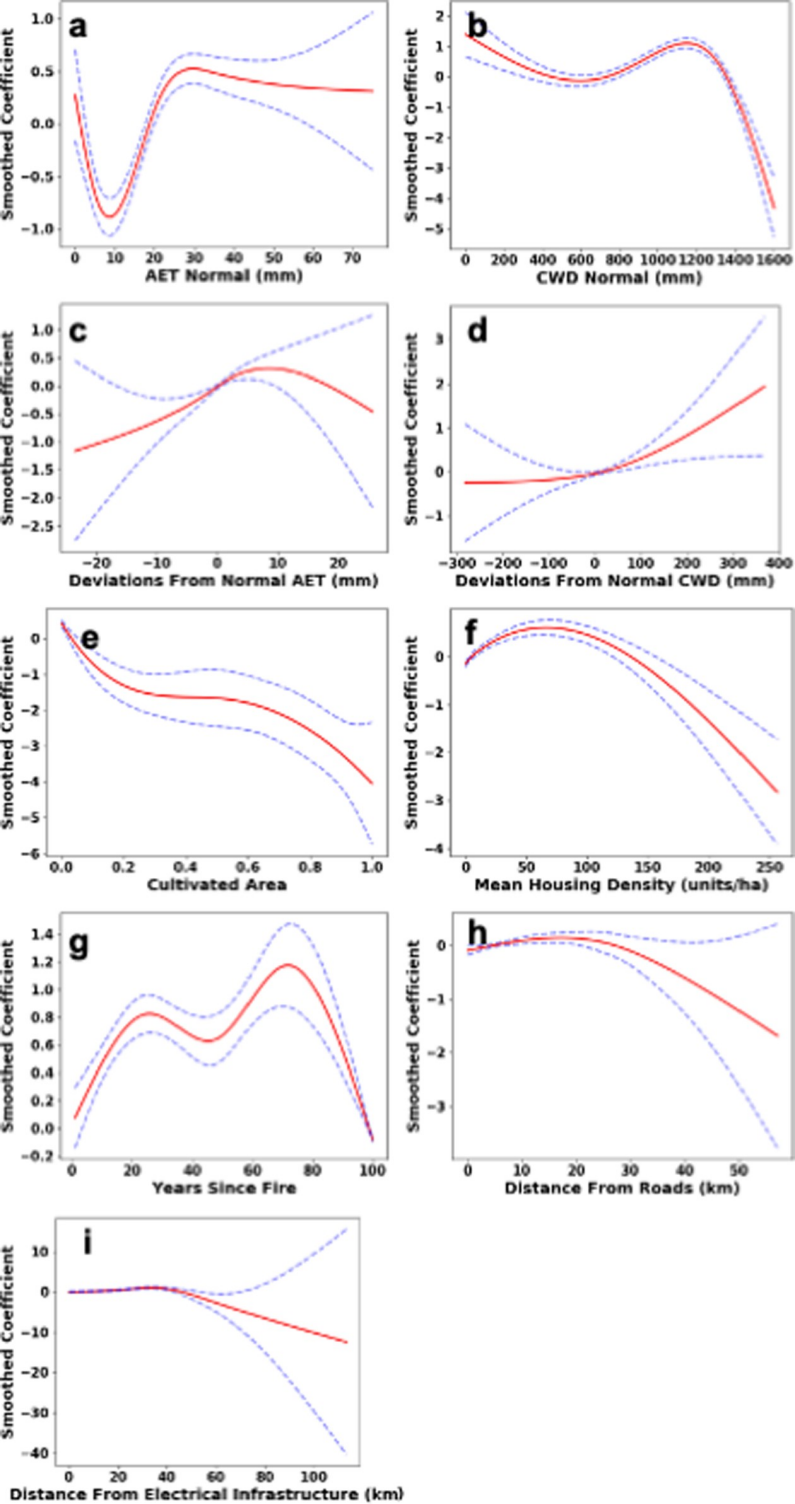
Smoothed coefficients for the statewide generalized additive model. Coefficients include (a) 1951–1980 normal actual evapotranspiration, (b) 1951–1980 normal climatic water deficit, (c) annual deviations from 1951–1980 normal actual evapotranspiration, (d) annual deviations from 1951–1980 normal climatic water deficit, (e) proportion of cultivated area, (f) annual mean housing density, (g) years since fire, (h) distance from roads, and (i) distance from electrical infrastructure.

Across California, both local climate and human activity contributed to observed patterns of fire. However, eliminating either climatic or anthropogenic factors resulted in significant shifts in predicted fire probabilities (Table 2, Figs 3 and 4). As hypothesized, the effects of human activity (housing density, percent cultivated area, and distances to roads or electrical infrastructure) were better predictors of local fire probability than local climate (r = 0.396 versus r = 0.302 for climate-only submodels, see Table 4). Furthermore, human activity on the landscape exhibited complex and sometimes antagonistic influences on fire probability. Notably, local housing density exhibited a complex relationship to fire that could either reduce or increase local fire probability depending on the level of development. In locations experiencing a high level of development (housing densities > 250 units/ha), greater housing density was strongly associated with reduced annual fire probability (Fig 5F), likely due to reduced fuel availability and increasingly effective fire management and suppression [6, 11, 66]. Similarly, low housing density was typically associated with reductions in annual fire probability (Fig 5F), likely due to fewer human-induced ignitions. In contrast, areas with moderate housing densities throughout the surrounding (25 km) area revealed higher annual fire probabilities (Fig 5F), likely due to a confluence of more frequent anthropogenic ignitions, greater fuel availability due to largely intact vegetation, and in some areas, limited accessibility to fire control personnel. These areas often reflect regions with scattered homes throughout the wildland-urban interface, as well as areas such as portions of the Southern California mountains such as the Angeles and Los Padres National Forests (Fig 1). Despite sometimes exhibiting few housing units within their boundaries, such regions are located in close proximity (within 25 km) to human activity and ignitions associated with the densely populated Los Angeles metropolitan area (S2D Fig). Distances from both primary or secondary roads and from electrical infrastructure exhibited a minimal relationship to historical fire probability, although fire probabilities were predicted to decrease among locations where distances from electrical infrastructure exceeded 45 km (Fig 5I, S2F Fig). In both cases, this pattern may be assumed to result from lower rates of anthropogenic ignitions among sites that were located at great distances from these forms of infrastructure [67]. Agricultural activity and cultivation, in contrast to other forms of human activity, was found to strongly reduce fire probability (Fig 5E). This effect was likely due to its association with irrigation, accessibility to fire control personnel, and thinning of more flammable vegetation. Collectively, these results agree with previous studies documenting the critical role that human activity plays in determining the frequency of fire across California [11, 68, 69] and that, across California, areas with intermediate population densities typically experience the highest probabilities of fire [6, 70, 71]. This study also highlights that various kinds and intensities of human activity may exert either positive or negative effects on the probability of fire.

### Effects of local climate on fire probability

Local climate conditions played a significant role in predicted fire probability throughout California (Table 2). AET normals exhibited a strong increase in fire probability as evapotranspiration shifted from low (<10 mm, Fig 5A) to moderate amounts (≥ 30 mm, Fig 5A), reflecting the effects of higher vegetative biomass (and therefore fuel availability), across densely vegetated areas such as the Sierra Nevada and Klamath ranges (Figs 1 and 5A, S2A Fig). It should be noted, however, that extremely low AET (AET <10) was associated with increases in fire probability; this pattern was driven almost exclusively by multiple fires throughout the western portion of California’s Central Valley (Fig 1), in which frequent large grass fires were observed throughout areas characterized by extremely low AET values. Exotic grasses often support far more frequent fire cycles than other vegetation types throughout the Western United States, and are likely to be the primary driver of this pattern [72, 73]. Our findings thus corroborate the hump-shaped response of fire activity to productivity first observed by Krawchuk et al. [74], which forms the basis of the global “varying constraints hypothesis” [1, 2]. However, the new peak of fire probabilities documented here, associated with “priming” of very unproductive environments by invasive grasses, may be an important new feature in the overall fire*productivity relationship.

In contrast to AET, CWD normals exhibited a positive effect on fire probability among locations that were characterized by moderate to high water deficits (>~600 mm—<~1100 mm). Across California, such locations typically experienced dry summer conditions that led to greater fuel flammability and resulting high fire probabilities [75]. However, higher CWD normals exhibited a negative effect among locations characterized by severe water deficits (>~ 1100mm). This pattern likely reflects the limited fuel availability among highly xeric locations, in which insufficient vegetation is present to sustain the spread of large fires (Fig 5B, S2B Fig).

Eliminating the effects of short-term (3 year) deviations from local climate normals did not significantly reduce overall model explanatory power when predicting mean annual fire probabilities over multidecadal timescales (Table 4). Nevertheless, short-term climate variations did have significant (Table 2) and systematic relationships to predicted annual fire probabilities throughout California (Fig 4). This apparent contradiction likely occurs because short-term climate variations in both AET and CWD impacted predictions of fire probability predominantly in those years that most deviated from local normal conditions (Fig 5C and 5D). Short-term climate deviations are most relevant to fire probabilities in those periods that represent extreme departures from local climate norms, and thus may not play a major role in determining fire probability except in years that experience extreme conditions. Three-year climate anomalies exhibited the most dramatic effect on fire probability in periods with higher than average CWD (Fig 5D), while years that exhibited unusually low CWD or AET also experienced moderate reductions in fire probability. When examining average fire probabilities across the years 1970–2016, the elimination of short-term climate deviations produced mild but systematic shifts in long-term fire probability over space. These shifts largely consisted of decreases in predicted fire probabilities throughout the transverse and peninsular ranges of southern California (Figs 1 and 4). Further, extreme climate events were found to affect annual probabilities throughout much of California (Fig 5C and 5D). This indicates that, while spatial variation in local climate normals and human activity throughout California may play larger roles in determining local fire probability throughout California, short-term variations in climate do play a significant role in the probability of fire in years with extreme conditions, particularly in certain fire-prone environments. Specifically, these results indicate that periods of extreme drought (i.e. years with unusually low CWD) are associated with greater annual fire probability throughout Southern California.

### Effects of time since fire on annual fire probability

Time since last fire also played a significant role in annual fire probability throughout California (Table 2). Annual fire probability increased rapidly throughout the first 20 years after a burn event (Fig 5G). This finding is consistent with previous studies that observed reduced ignition and spread of wildfire in recently burned portions of the Rocky Mountains [32]. The rapid restoration of annual fire probability beginning immediately post-fire likely occurs because, in many fire-prone ecosystems within California, fire-associated tree mortality can be low [76], and surface fuels such as grasses or surface litter regenerate rapidly after fire. However, resulting fire sizes may be limited in the years immediately following fires [33], and the actual strength and duration of post-fire reductions in annual fire probability are likely to vary among vegetation types and by the rate at which fuels regenerate in a given location [32]. Fire probability was found to be highest approximately 80 years post fire, likely due to a progressive accumulation of fuel. As the limited duration of fire records throughout California limited the maximum observed time since fire to 100 years (i.e. time since fire was always set to a maximum value of 100 years), there is likely some conflation of locations which have not been observed to burn within the past 100 years but may burn, and highly xeric or barren locations in which no fire has likely occurred in thousands of years due to a lack of sufficient fuel to carry a wildfire. As a result of this data limitation, this model predicts reduced fire probability in locations in which fire has not occurred for 100 years or more. Additionally, as previous studies have found that previous fires provide barriers to subsequent fire events for varying lengths of time across different vegetation types [33, 34, 77], it is likely that some additional interactions between time since fire and local vegetation may be overlooked in this study due to a lack of vegetation maps that track historical vegetation at annual timescales. Nevertheless, the observed reductions in probability of fire in the years following a prior fire event emphasized the importance of the short-term temporal aspect of fire load on wildfire across California, and the potential for previous burn events to act as potential barriers to fire. This pattern also indicated that management practices such as controlled burns or fuel reductions, if conducted safely and at frequent intervals, could significantly reduce wildfire probabilities throughout much of California.

### Modeling fire probability at statewide versus regional scales

The statewide model systematically outperformed regional models in successfully distinguishing between areas of high and low fire probability (Table 3). Further, predictions of mean annual fire probability produced by regional models demonstrated no significant correlation to observed historical fire probabilities within the region in which they were trained (r <0.01, p = 0.11 among regional models [excluding the Sonoran and Mojave Desert regions in which an insufficient number of fires were observed to produce regional models, Figs 1 and 2C], versus r = 0.59, p<0.01 among statewide models). While relationships between each parameter and predicted fire probabilities were not always consistent among regions, this appeared to largely be the result of either limited variation in a given parameter within a region (e.g. climatic homogeneity), or of overfitting on individual large fire events due to limited number of fires observed within some regions. Furthermore, regional models have previously been observed to perform poorly when applied to conditions which did not overlap with those in which they were trained [42]. In this study, this effect was primarily observed near the borders between adjacent regions, where local conditions were often different from the remainder of that region, and in which predicted fire probabilities produced by regional models were therefore sometimes wildly inaccurate. Thus, while the contributions and relative importances of local conditions may vary with the status of local conditions over space [7, 42, 69], this study demonstrates that, when working at 1-km spatial resolution, localized modeling of fire probability is not necessarily desirable or preferable to broad-scale modeling. When using a nonlinear modeling framework that is capable of adapting to differing relationships between local conditions and the resulting fire regime, localized or regional modeling approaches may limit model transferability to other regions [42], and by extension, transferability to new conditions within that region. Further, this study indicates that regional models underperform broad-scale models even within their own boundaries due to their reduced quantity of training data, greater potentiality for edge effects, and increased potential for overfitting to specific fire events. In contrast, broad-scale state-wide or multi-region models benefit from the incorporation of a wider range of conditions, a greater number of historical fire events, and fewer artificial disjuncts in predicted fire probability. Further, broad-scale models demonstrate high predictive ability among both novel locations and novel years (Table 3). As computational power and the scope of spatially explicit data continue to increase, these results emphasize the power of large-scale machine learning techniques to provide powerful, holistic models of fire probability. It should be noted, however, that while broad-scale models may outperform regional models at 1-km resolution, finer-scale models of fire behavior, which are more sensitive to subtle variations in local conditions, may still require highly localized modeling frameworks. Additionally, this dataset only examines timber fires of 10+ acres (0.04 km^2^), brush fires of 30+ acres (0.12 km^2^), and grass fires of 300+ acres (1.21 km^2^). Thus, this dataset likely overlooks many small fires that were rapidly contained or failed to spread sufficiently to be included in FRAP records, and for which finer-scale analysis might be required.

### Comparison to competing models

Our findings corroborate generalized constraints on fire activity reviewed in Krawchuk and Moritz [1], as well as prior studies by Syphard [19] and Mann [11] in which local climate and human activity were observed to play critical roles in determining the local probability of fire throughout California. However, this method exhibits several advantages over previous methods. For instance, our model derived the response curve of fire probability to each parameter directly from the historical data, rather than restricting those responses to linear [11, 19], log [78], or predetermined polynomial orders [11]. In contrast, GAM response curves are based on multiple smooths rather than the parameterization of preset curve types. The model used in this study was therefore far less restricted in its ability to model nonlinear responses. By generating these response curves directly, this method provides unique insights into the contributions of human activity and local climate to the resulting fire probabilities both throughout the observed range of each parameter (Fig 5) and across California (S2 Fig). Importantly, the high cross-validated ROC/AUC scores exhibited by models produced in this study, as well as the strong correlations between predicted and observed fire frequencies (Tables 3 and 4) indicate that these curves meaningfully predict actual fire probabilities and do not suffer from overfitting.

## Conclusions

This study demonstrates that local climate–through limitations posed by fuel dryness (CWD) and fuel availability (AET)–plays an important and predictable role in determining the annual probabilities of fire throughout California. Further, our findings emphasize the importance of incorporating human activity–through influences on ignitions and suppression of fires–into predictions of fire probability over space and time. We also document the importance of previous burn events as potential barriers to fire in some environments, until enough time has passed for vegetation to regenerate sufficiently to sustain wildfire events. While confirming previous findings that human activity is critical for fire prediction, this study also demonstrates that, although interannual climate variation typically only exhibits significant impacts of fire probability in years that undergo extreme conditions, such changes can be an important aspect of fire probability, particularly in certain fire-prone regions such as the southern California shrublands and forests. Further, it demonstrates a novel methodology for applying the varying constraints framework to fire probability modeling that is simultaneously capable of producing powerful estimates of fire probability while also illuminating the relationship of local climate and human activity on spatiotemporal patterns of fire. Finally, this study represents a powerful tool for mapping local fire probability across the state of California under a variety of historical climate regimes, which is essential to avoided emissions modeling, carbon accounting, and hazard severity mapping for the application of fire-resistant building codes across the state of California. As these methods advance and additional data becomes available, these techniques may be further refined to examine the effects of historical and projected changes in vegetation on resulting fire return intervals, to predict future patterns of fire under specific climate change and development scenarios, to conduct finer-scale assessments of the impacts of specific forms of human activity or development on local fire probability or hazard, or to incorporate the effects of additional parameters such as live fuel moisture on resulting fire probability, size, or burn severity.

## Supporting information

S1 Fig Study area (a) and example distribution of pixels selected using Poisson disk regularization with a 5-km minimum distance between pixels (b). Red squares correspond to selected pixels.
(TIF)

S2 Fig Smoothed Coefficients of a) 1951–1980 normal actual evapotranspiration, b) 1951–1980 climatic water deficit, c) proportion of cultivated area, d) mean housing density (over the years 1970–2016), e) distance from roads, f) distance from electrical infrastructure, and g) mean time since last fire across California, from statewide GAM model.
(TIF)

S1 Table Distribution of pixels selected by Poisson-disk regularization among individual fire events across different minimum distance thresholds. (DOCX)

## References

[1] Krawchuk MA, Moritz MA. Constraints on global fire activity vary across a resource gradient. Ecology. 2011;92(1):121–32. doi: 10.1890/09-1843.1 21560682

[2] Krawchuk MA, Moritz MA. Burning issues: statistical analyses of global fire data to inform assessments of environmental change. Envirometrics. 2014;25(6):472–81.

[3] Moritz MA, Parisien M-A, Batllori E, Krawchuk MA, Van Dorn J, Ganz DJ. Climate change and disruptions to global fire activity. Ecosphere. 2012;3(6):1–22.

[4] Parks SA, Miller C, Nelson CR, Holden ZA. Previous Fires Moderate Burn Severity of Subsequent Wildland Fires in Two Large Western US Wilderness Areas. Ecosystems. 2014;17(1):29–42.

[5] Westerling AL, Bryant BP. Climate change and wildfire in California. Climate Change. 2008;87:S231–S49.

[6] Syphard AD, Radeloff VC, Keeley JE, Hawbaker TJ, Clayton MK, Stewart SI, et al. Human influence on California fire regimes. Ecological Applications. 2007;17(5):1388–402. doi: 10.1890/06-1128.1 17708216

[7] Bistinas I, Oom D, Sá ACL, Harrison SP, Prentice IC, Pereira JMC. Relationships between human population density and burned area at continental and global scales. PLOS ONE. 2013;8(12):e81188. doi: 10.1371/journal.pone.0081188 24358108 PMC3865302

[8] Balch JK, Bradley BA, Abatzoglou JT, Nagy RC, Fusco EJ, Mahood AL. Human-started wildfires expand the fire niche across the United States. Proceedings of the National Academy of Sciences. 2017;114(11):2946–51. doi: 10.1073/pnas.1617394114 28242690 PMC5358354

[9] Fusco EJ, Abatzoglou JT, Balch JK, Finn JT, Bradley BA. Quantifying the human influence on fire ignition across the western USA. Ecological Applications. 2016;26(8):2390–401. doi: 10.1002/eap.1395 27907256

[10] Keeley JE, Syphard AD. Historical patterns of wildfire ignition sources in California ecosystems. International Journal of Wildland Fire. 2018;27(12):781–99.

[11] Mann ML, Batllori E, Moritz MA, Walker EK, Berck P, Flint AL, et al. Incorporating anthropogenic influences into fire probability models: effects of human activity and climate change on fire activity in California. Plos One. 2016;11(4):e0153589. doi: 10.1371/journal.pone.0153589 27124597 PMC4849771

[12] Guyette RP, Muzika RM, Dey DC. Dynamics of an Anthropogenic Fire Regime. Ecosystems. 2002;5(5):472–86.

[13] Butsic V, Kelly M, Moritz MA. Land use and wildfire: a review of local interactions and teleconnections. Land. 2015;4:140–56.

[14] Pausas JG, Paula S. Fuel shapes the fire–climate relationship: evidence from Mediterranean ecosystems. Global Ecology and Biogeography. 2012;21(11):1074–82.

[15] Schoennagel T, Veblen TT, Romme WH. The Interaction of Fire, Fuels, and Climate across Rocky Mountain Forests. BioScience. 2004;54(7):661–76.

[16] Wotton BM, Flannigan MD. Length of the fire season in a changing climate. The Forestry Chronicle. 1993;69(2).

[17] Yoon J-H, Wang S-YS, Gillies TR, Hipps L, Kravitz B, Philip R. Extreme fire season in California: a glimpse into the future? Bulletin of the American Meteorological Society. 2015;96(12):5–9.

[18] Goss M, Swain DL, Abatzoglou JT, Sarhadi A, Kolden CA, Williams AP, et al. Climate change is increasing the likelihood of extreme autumn wildfire conditions across California. Environmental Research Letters. 2020;15(9):094016.

[19] Syphard AD, Rustigian-Romsos H, Mann M, Conlisk E, Moritz MA, Ackerly D. The relative influence of climate and housing development on current and projected future fire patterns and structure loss across three California landscapes. Glob Environ Change-Human Policy Dimens. 2019;56:41–55.

[20] Chou YH, Minnich RA, Chase RA. Mapping probability of fire occurrence in San Jacinto Mountains, California, USA. Environmental Management. 1993;17(1):129–40.

[21] Fried JS, Torn MS, Mills E. The impact of climate change on wildfire severity: a regional forecast for Northern California. Climatic Change. 2004;64:169–91.

[22] Huang Y, Yufang J, Schwartz MW, Thorne JH. Intensified burn severity in California northern coastal mountains by drier climatic condition. Environmental Research Letters. 2020;15.

[23] Kayser A, Westerling AL. Predicting increasing high severity area burned for three forested regions in the western United States using extreme value theory. For Ecol Manage. 2019;432:694–706.

[24] Keyser AR, Westerling AL. Predicting increasing high severity area butned for three forested regions in the western United States using extreme value theory. For Ecol Manage. 2019;432:694–706.

[25] Kane VR, Cansler CA, Povak NA, Kane JT, McGaughey RJ, Lutz JA, et al. Mixed severity fire effects within the Rim fire: Relative importance of local climate, fire weather, topography, and forest structure. For Ecol Manage. 2015;358:62–79.

[26] Yang J, Weisberg PJ, Dilts TE, Loudermilk EL, Scheller RM, Stanton A, et al. Predicting wildfire occurrence distribution with spatial point process models and its uncertainty assessment: a case study in the Lake Tahoe Basin, USA. International Journal of Wildland Fire. 2015;24(3):380–90.

[27] Littell JS, Gwozdz RB. Climatic Water Balance and Regional Fire Years in the Pacific Northwest, USA: Linking Regional Climate and Fire at Landscape Scales. In: McKenzie D, Miller C, Falk DA, editors. The Landscape Ecology of Fire. Dordrecht: Springer Netherlands; 2011. p. 117–39.

[28] Trouet V, Taylor AH, Wahl ER, Skinner CN, Stephens SL. Fire-climate interactions in the American west since 1400 CE. Geophysical Research Letters. 2010;37.

[29] Jin Y, Randerson JT, Faivre N, Capps S, Hall A, Goulden ML. Contrasting controls on wildland fires in Southern California during periods with and without Santa Ana winds. Journal of Geophysical Research: Biogeosciences. 2014;119:432–50.

[30] Fry DL, Stephens SL. Influence of humans and climate on the fire history of a ponderosa pine-mixed conifer forest in the southeastern Klamath Mountains, California. For Ecol Manage. 2006;223(1):428–38.

[31] Littell JS. Drought and Fire in the western USA: Is climate attribution enough? Current Climate Change Reports. 2018;4:396–406.

[32] Parks SA, Miller C, Holsinger LM, Baggett LS, Bird BJ. Wildland fire limits subsequent fire occurrence. International Journal of Wildland Fire. 2016;25:182–90.

[33] Parks SA, Holsinger LM, Miller C, Nelson CR. Wildland fire as a self-regulating mechanism: the role of previous burns and weather in limiting fire progression. Ecological Applications. 2015;26(6):1478–92. doi: 10.1890/14-1430.1 26552258

[34] Collins BM, Miller JD, Thode AE, Kelly M, van Wagdetonk JW, Stephens SL. Interactions among wildland fires in a long-established Sierra Nevada natural fire area. Ecosystems. 2009;12:114–28.

[35] Minnich RA. An Integrated Model of Two Fire Regimes. Conservation Biology. 2001;15(6):1549–53.

[36] Littell JS, McKenzie D, Peterson DL, Westerling AL. Climate and wildfire area burned in western U.S. ecoprovinces, 1916–2003. Ecological Applications. 2009;19(4):1003–21. doi: 10.1890/07-1183.1 19544740

[37] Moritz MA, Keeley JE, Johnson EA, Schaffner AA. Testing a basic assumption of shrubland fire management: how important is fuel age? Frontiers in Ecology and the Environment. 2004;2(2):67–72.

[38] Tiribelli F, Kitzberger T, Morales JM. Changes in vegetation structure and fuel characteristics along post-fire succession promote alternative stable states and positive fire–vegetation feedbacks. Journal of Vegetation Science. 2018;29(2):147–56.

[39] Eskelon BNI, Monleon VJ. Post-fire surface fuel dynamics in California forests across three burn severity classes. International Journal of Wildland Fire. 2018;27:114–24.

[40] Keeley JE, Syphard AD. Climate change and future fire regimes: Examples from California. geosciences. 2016;6(37).

[41] Keeley JE, Syphard AD. Different historical fire-climate patterns in California. International Journal of Wildland Fire. 2017;26:253–68.

[42] Parisien M-A, Moritz MA. Environmental controls on the distribution of wildfire at multiple spatial scales. Ecological Monographs. 2009;79(1):127–54.

[43] Krawchuk MA, Moritz MA. Fire and Climate Change in California. In: Commission CE, editor.: Simon Fraser University and University of California, Berkeley; 2012.

[44] Keeley JE. Impact of antecedent climate on fire regimes in coastal California. International Journal of Wildland Fire. 2004;13(2):173–82.

[45] McClellan C. CALVEG, [ESRI personal geodatabase]. In: USDA-Forest Service PSR, editor. 2008.

[46] Flint LE, Flint AL, Stern MA. The Basin Characterization Model—A regional water balance software package (BCMv8) data release and model archive for hydrologic California, water years 1896–2020. 2021.

[47] Flint LE, Flint AL, Stern MA. The Basin Characterization Model version 8 –A Regional Water Balance Code Calibration and Application. US Geological Survey Techniques and Methods. 2021 (In Press).

[48] Group PC. http://prism.oregonstate.edu. PRISM Climate Group: Oregon State University; Feb 2 2004.

[49] Flint L, Flint A, Thorne J, Boynton T. California Basin Characterization Model Downscaled Climate and Hydrology. In: Flint L, editor. Reston, VA: U.S. Geological Survey; 2013.

[50] Yang L, Jin S, Danielson P, Homer C, Gass L, Case A, et al. A New Generation of the United States National Land Cover Database: Requirements, Research Priorities, Design, and Implementation Strategies. ISPRS Journal of Photogrammetry and Remote Sensing. 2018;146:108–23.

[51] (EPA) USEPA. Land use scenarios: National-scale housing density scenarios consistent with climate change storylines. National Center for Environmental Assessment, Washington, DC; 2009.

[52] 2018 TIGER/Line roads. 2018.

[53] Thong FF. Electrical Transmission Lines [ds1198]. In: Commission CE, editor. Sacramento, CA: California Energy Commission; 2020.

[54] FRAP. Fire Perimeter Project Introduction. In: Fire C, editor. https://frap.fire.ca.gov/media/10969/fire19_1.zip2019.

[55] Van Rossum G, Drake FL. Python 3 Reference Manual. Scotts Valley, CA: CreateSpace; 2009.

[56] Homer CG, Dewitz Ja, Yang L, Jin S, Danielson P, Xian G, et al. Completion of the 2011 national land cover database for the conterminous United States—Representing a decade of land cover change information. Photogrammetric Engineering & Remote Sensing. 2015;81(5):345–54.

[57] McCool M, editor Hierarchical Poisson Disk Sampling Distributions. Proceedings of the conference on Graphics interface ’92; 1992.

[58] Wood SN. Fast stable restricted maximum likelihood and marginal likelihood estimation of semiparametric generalized linear models. Journal of the Royal Statistical Society (B). 2011;73:3–36.

[59] Bolopolsky A, Chapman B, Cock P, Eddelbuettel D, Kluyver T, Moreira W, et al. rpy2 2.9.4 documentation 2020 [Available from: https://rpy2.github.io/doc/v2.9.x/html/overview.html.

[60] Hand DJ, Till RJ. A simple generalization of the Area under the ROC curve for multiple class classification problems. Machine Learning. 2001;45:171–86.

[61] Sokolova M, Japkowicz N, Szpakowicz S, editors. Beyond Accuracy, F-Score and ROC: A Family of Discriminant Measures for Performance Evaluation2006; Berlin, Heidelberg: Springer Berlin Heidelberg.

[62] Benedetti R. Scoring rules for forecast verification. Monthly Weather Review. 2010;138(1):203–11.

[63] Wilks DS. Sampling distributions of the Brier score and Brier skill score under serial dependence. Quarterly Journal of the Royal Meteorological Society. 2010;136(653):2109–18.

[64] Weiss GM. Foundations of Imbalanced Learning. In: He H, Ma Y, editors. Imbalanced Learning: Foundations, Algorithms, and Applications. Hoboken, New Jersey: John Wiley & Sons; 2013.

[65] Hunter JD. Matplotlib: A 2D graphics environment. Computing in Science & Engineering. 2007;9(3):90–5.

[66] Lampin-Maillet C, Jappiot M, Long M, Bouillon C, Morge D, Ferrier J-P. Mapping wildland-urban interfaces at large scales integrating housing density and vegetation aggregation for fire prevention in the South of France. J Environ Manage. 2010;91(3):732–41. doi: 10.1016/j.jenvman.2009.10.001 19879685

[67] Syphard AD, Keeley JE. Location, timing and extent of wildfire vary by cause of ignition. International Journal of Wildland Fire. 2015;24(1):37–47.

[68] Faivre N, Jin Y, Goulden ML, Randerson JT. Controls on the spatial pattern of wildfire ignitions in Southern California. International Journal of Wildland Fire. 2014;23(6):799–811.

[69] Parisien M-A, Miller C, Parks SA, DeLancey ER, Robinne F-N, Flannigan MD. The spatially varying influence of humans on fire probability in North America. Environmental Research Letters. 2016;11(7):18.

[70] Parisien M-A, Snetsinger S, Greenberg JA, Nelson CR, Schoennagel T, Dobrowski S, et al. Spatial variability in wildfire probability across the western United States. International Journal of Wildland Fire. 2012;21:313–27.

[71] Mansuy N, Miler C, Parisien M-A, Parks SA, Batllori E. Contrasting human influences and macro-environmental factors on fire activity inside and outside protected areas of North America. Environmental Research Letters. 2019;14(6):12.

[72] Balch JK, Bradley BA, D’Antonio CM, Gómez-Dans J. Introduced annual grass increases regional fire activity across the arid western USA (1980–2009). Global Change Biology. 2013;19(1):173–83. doi: 10.1111/gcb.12046 23504729

[73] D’Antonio CM, Vitousek PM. Biological invasions by exotic grasses, the grass/fire cycle, and global change. Annu Rev Ecol Syst. 1992;23:63–87.

[74] Krawchuk MA, Moritz MA, Parisien M-A, Van Dorn J, Hayhoe K. Global pyrogeography: the current and future distribution of wildfire. Plos One. 2009;4(4):e5102. doi: 10.1371/journal.pone.0005102 19352494 PMC2662419

[75] Littell JS, Peterson DL, Riley KL, Liu Y, Luce CH. A review of the relationships between drought and forest fire in the United States. Global Change Biology. 2016;22:2353–69. doi: 10.1111/gcb.13275 27090489

[76] Stephens SL, Moghaddas JJ. Silvicultural and reserve impacts on potential fire behavior and forest conservation: Twenty-five years of experience from Sierra Nevada mixed conifer forests. Biological Conservation. 2005;125(3):369–79.

[77] Miller JD, Skinner CN, Safford HD, Knapp EE, Ramirez CM. Trends and causes of severity, size, and number of fires in northwestern California, USA. Ecological Applications. 2012;22(1):184–203. doi: 10.1890/10-2108.1 22471083

[78] Keyser A, Westerling AL. Climate drives inter-annual variability in probabiliity of high severity fire occurrence in the western United States. Environmental Research Letters. 2017;12(6). doi: 10.1088/1748-9326/aa7146 30344619 PMC6192430

