# Supplementary figures and images for: Relationships of climate, human activity, and fire history to spatiotemporal variation in annual fire probability across California
Source: PLoS One. 2021 Nov 3;16(11):e0254723. doi: 10.1371/journal.pone.0254723 (PMC8565767; doi:10.1371/journal.pone.0254723)

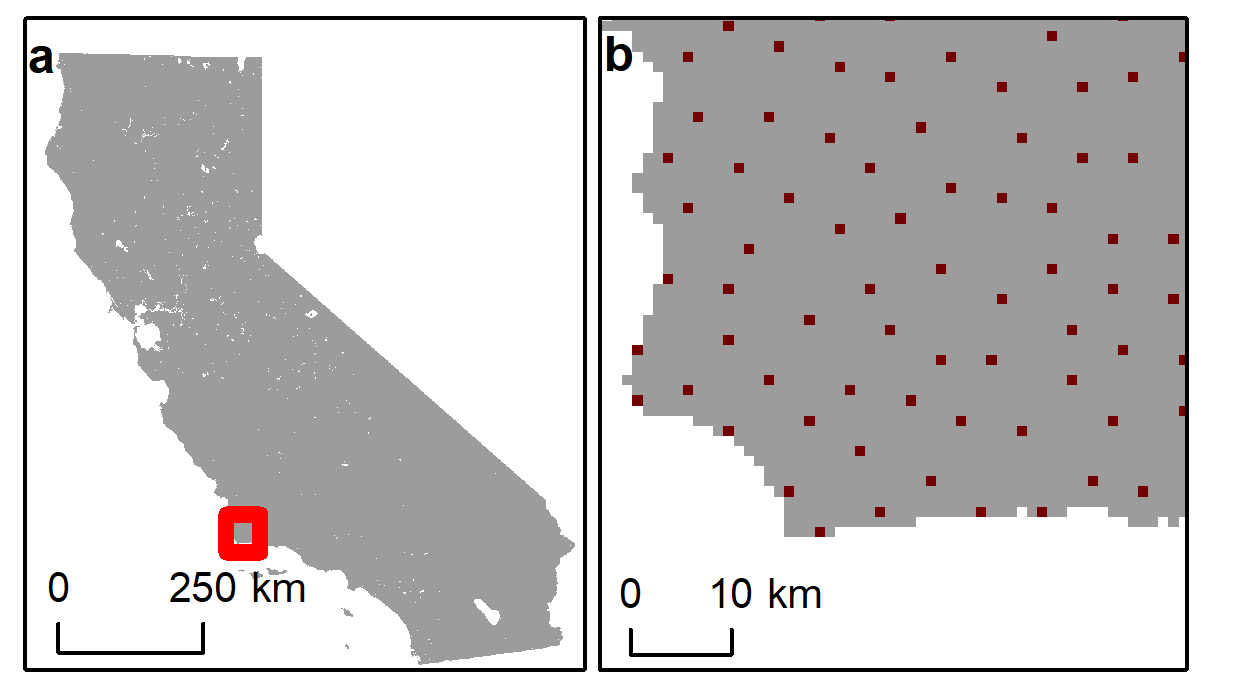

Supplement: S1 Fig — Study area (a) and example distribution of pixels selected using Poisson disk regularization with a 5-km minimum distance between pixels (b). Red squares correspond to selected pixels. (TIF) [file pone.0254723.s001.tif]

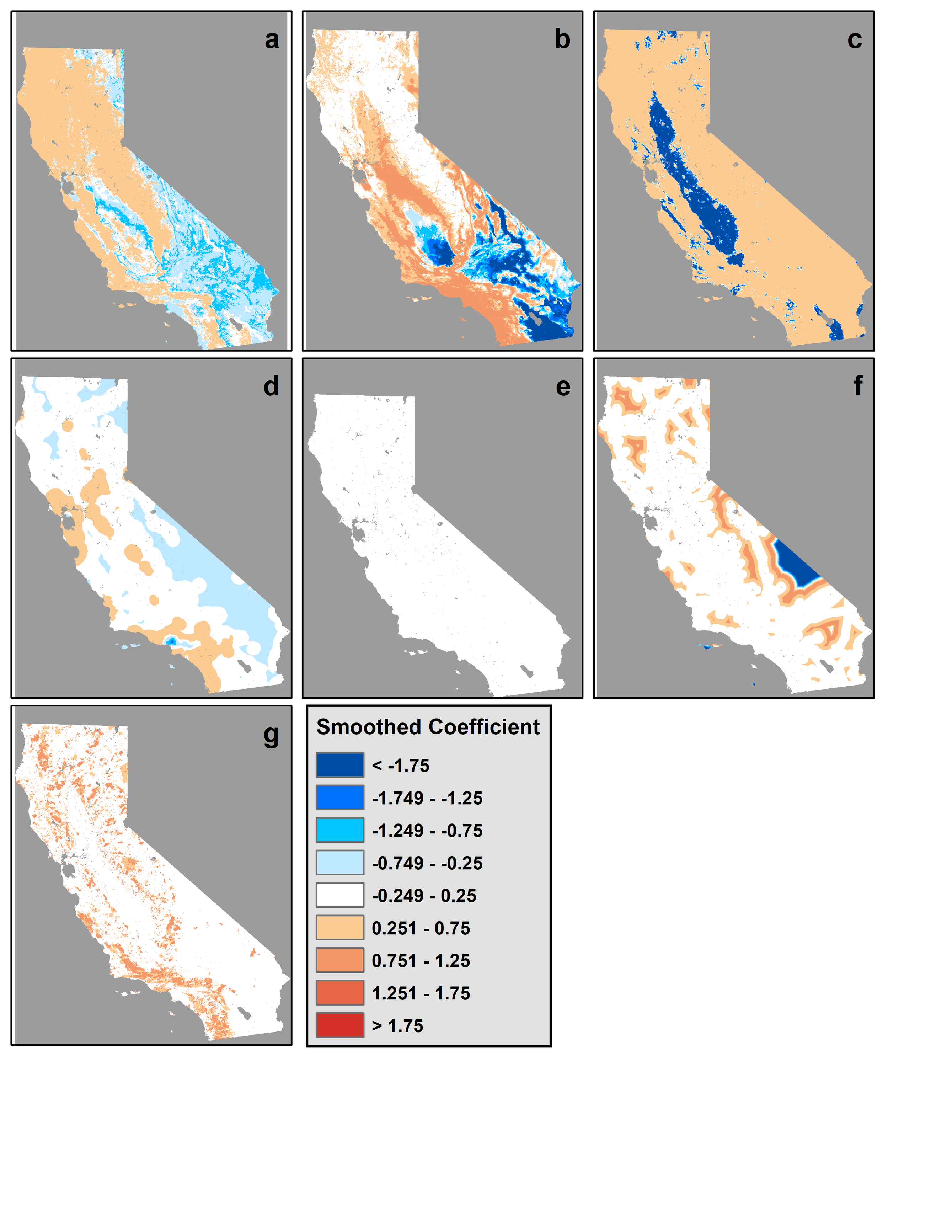

Supplement: S2 Fig — Smoothed Coefficients of a) 1951–1980 normal actual evapotranspiration, b) 1951–1980 climatic water deficit, c) proportion of cultivated area, d) mean housing density (over the years 1970–2016), e) distance from roads, f) distance from electrical infrastructure, and g) mean time since last fire across California, from statewide GAM model. (TIF) [file pone.0254723.s002.tif]
