# Supplementary material for: Relationships of climate, human activity, and fire history to spatiotemporal variation in annual fire probability across California
Source: PLoS One. 2021 Nov 3;16(11):e0254723. doi: 10.1371/journal.pone.0254723 (PMC8565767; doi:10.1371/journal.pone.0254723)
Supplement: S1 Table — (DOCX) [file pone.0254723.s003.docx]

| **Distance Threshold (km)** | **# Pixels** | **# Burned Pixels** | **# Unique Fires** | **# pixels in which a unique fire is sampled X times** | | | | | |
| --- | --- | --- | --- | --- | --- | --- | --- | --- | --- |
|  |  |  |  | **1** | **2** | **3** | **4** | **5** | **10+** |
| 2 | 3,054295 | 11,916 | 2,637 | 1,498 | 754 | 537 | 404 | 430 | 7,202 |
| 5 | 493,876 | 1,914 | 948 | 672 | 230 | 156 | 108 | 100 | 372 |
| 10 | 126,148 | 515 | 397 | 334 | 70 | 39 | 36 | 10 | 0 |
